# Hyoid Elongation May Be a Rare Cause of Recurrent Ischemic Stroke in Youth-A Case Report and Literature Review

**DOI:** 10.3389/fneur.2021.653471

**Published:** 2021-09-01

**Authors:** Gang Liu, Yuan Wang, Changbiao Chu, Yi Ren, Yang Hua, Xunming Ji, Haiqing Song

**Affiliations:** ^1^Department of Neurology, Xuanwu Hospital, Capital Medicine University, Beijing, China; ^2^Department of Vascular Ultrasound, Xuanwu Hospital, Capital Medical University, Beijing, China; ^3^Department of Neurosurgery, Xuanwu Hospital, Capital Medicine University, Beijing, China

**Keywords:** recurrent stroke, young adults, hyoid bone, carotid artery diseases, imaging diagnosis

## Abstract

The investigation for etiology of ischemic stroke in young adults remains a diagnostic challenge. Hyoid bone–related carotid injury is a rare and under-recognized cause of ischemic stroke, without established guidelines. We describe a case of recurrent ischemic stroke in a young patient presumably attributed to an impingement of the carotid artery by an elongated hyoid bone, and present other cases reported in the literature. Based on the imaging study as well as the lack of other findings, we attributed recurrent neurovascular events to the repetitive mechanical stimulation by the elongated hyoid bone that caused a vessel wall injury with subsequent thrombus and embolus. Given repeated recurrence under antiplatelet treatment, anticoagulation was added. The following 2-year follow-up showed no new neurologic events or any other complaints. Among the young, a broad spectrum of possibilities should be considered and we call attention to this infrequent etiology of ischemic stroke.

## Background

Every year, more than two million young adults experience an ischemic stroke worldwide. Stroke at young ages has been considered as an enormous socio-economic problem due to high health-care costs and loss of productivity (1). By contrast to stroke in the elders, stroke in young adults is more heterogeneous because of a wide spectrum of possible underlying risk factors and often rare etiologies (2). Meanwhile, investigations into the cause of ischemic stroke at a young age is often challenging.

As an uncommon etiology of stroke, hyoid bone elongation causes compression or localized trauma to the carotid artery. We describe a young patient with recurrent strokes resulting from mechanical interference of an elongated hyoid bone to the carotid artery. Previous reported cases from 1999 to present are also summarized (Supplementary Table 1).

## Case Presentation

A 39-year-old male patient was admitted to the Neurology Department with the complaint of dysphasia with a sudden onset 2 years ago and sudden right limb weakness for 4 months, who had the habit of playing badminton and golf.

Two years ago, the patient experienced intermittent episodes of difficulty in speaking and language comprehension with resolution after about 2 h. Head magnetic resonance imaging (MRI) showed abnormal signal intensity in the left temporal lobe, indicating acute infarction, while head magnetic resonance angiography (MRA) was normal. The transesophageal echocardiogram (TEE) combined with color transcranial doppler (TCD) indicated paradoxical embolism from a patent foramen ovalis (PFO). He was diagnosed as cerebral infarction (left temporal lobe) with high possibility of cardiac embolism and PFO. Then, he underwent transcatheter PFO closure successfully. After that, he adhered to aspirin (100 mg once per day) and atorvastatin (10 mg once per day) therapy.

Four months before admission, the patient experienced weakness of right limbs and inability to speak with a sudden onset, and recovered within seconds spontaneously. The next day, he was noted by his colleagues due to difficulty speaking accompanied by low spirits, and was sent to the local hospital, where he was diagnosed as acute cerebral infarction. At that time, his head MRI revealed acute infarction on diffusion-weighted imaging (DWI) sequences in the left anterior cerebral artery (ACA) and middle cerebral artery (MCA) territory. The digital subtraction angiography (DSA) revealed M1 segment occlusion of the left MCA. He underwent endovascular therapy (thrombectomy) of the left proximal MCA, and recovered with sequelae of mild dysarthria and poor short-term memory. He was then discharged with a prescribed regimen of aspirin (100 mg once per day) and atorvastatin (10 mg once per day).

**Figure 1:**
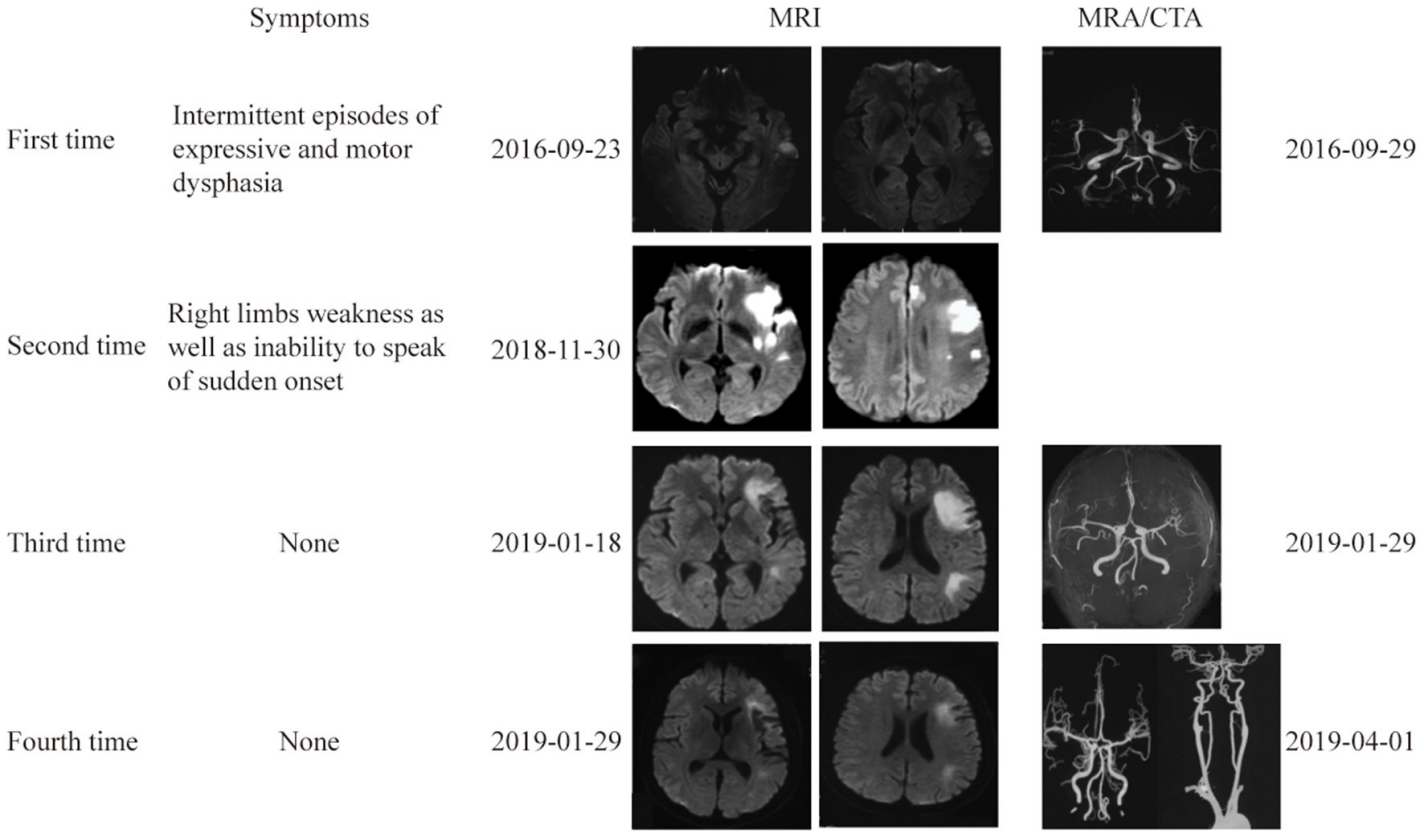
Brain and vascular imaging at 4 different times. Brain MRI at 4 times showed multiple lesions in the left ICA territory. Vascular imaging showed no evidence of vascular stenosis or occlusion.

**Figure 2:**
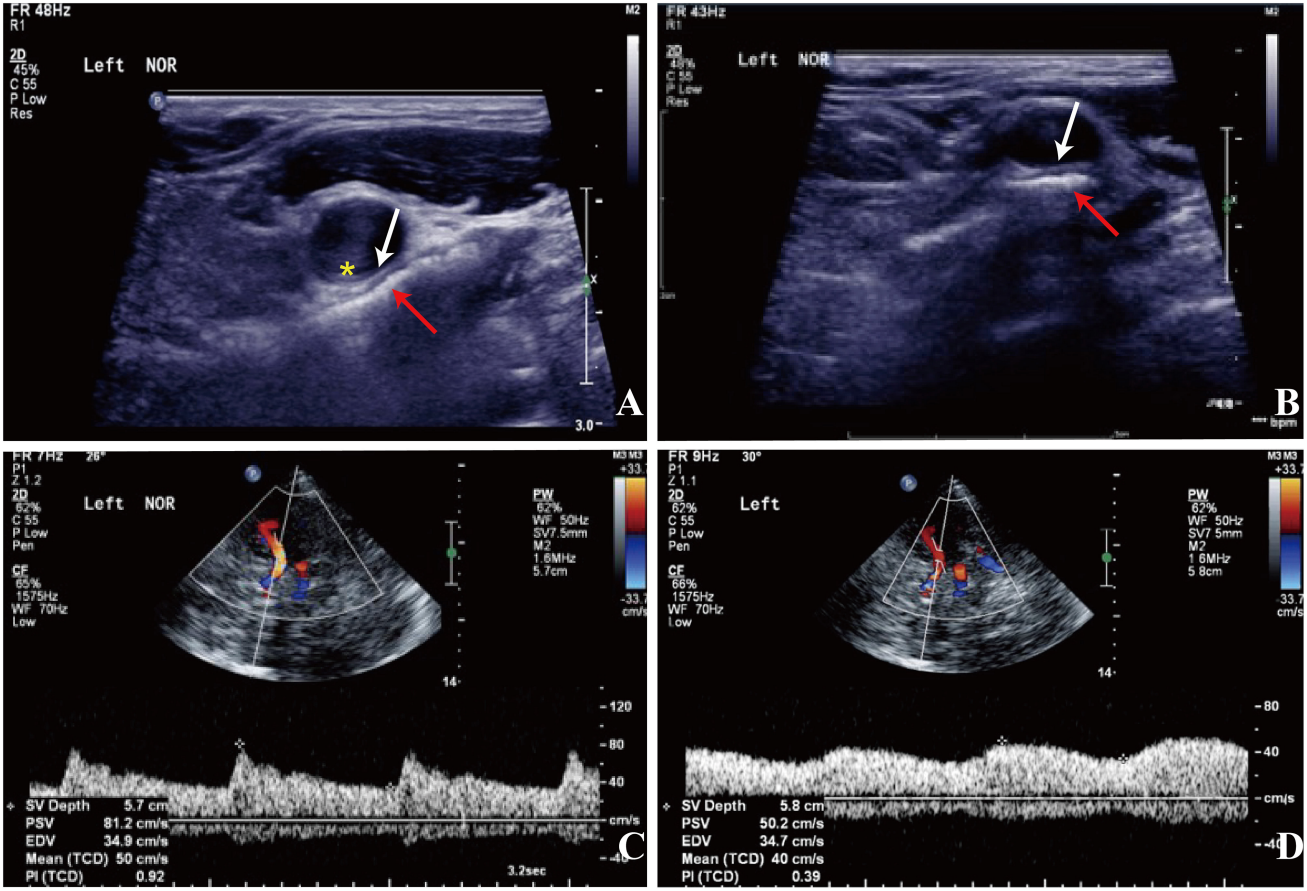
Carotid doppler ultrasound spectrum (DUS) **(A,B)** and TCCD **(C,D)**. **(A,B)** There was a heterogeneous echo outside the left carotid bifurcation, and the lumen at the distal end of the left CCA was compressed by the hyoid bone in the rotated neck position (red arrow: the hyoid bone; white arrow: compressed arterial lumen; yellow asterisk: increased IMT). **(C)** The flow velocity of the left MCA was normal in the neutral position. **(D)** The flow velocity of the left MCA decreased significantly with neck rotation.

**Figure 3:**
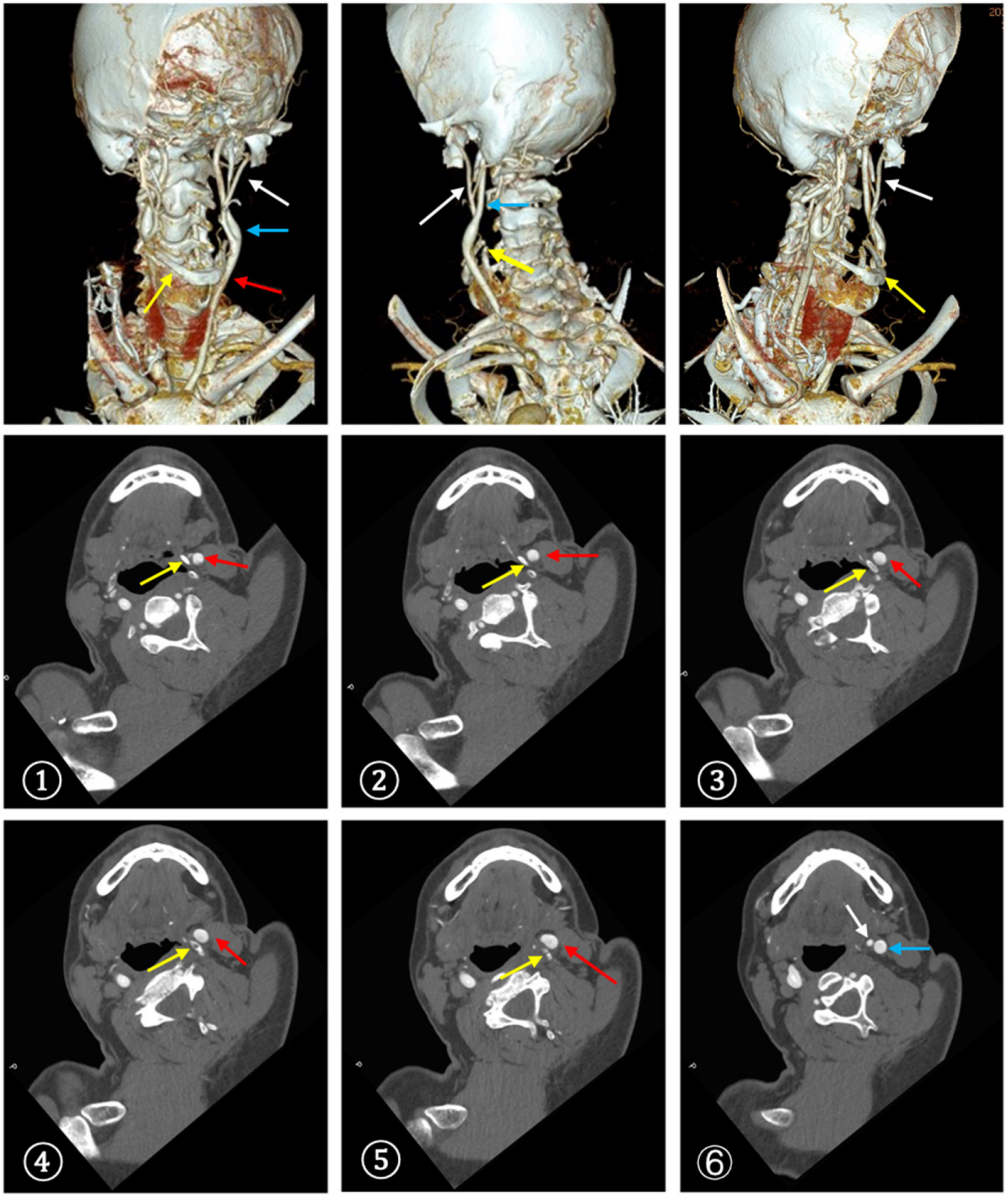
CTA demonstrated a close contact between the enlarged left greater horn of the hyoid bone and the carotid bifurcation. -, The serial CT slices from distal CCA to proximal ICA. (red arrow: CCA, blue arrow: ICA, white arrow: ECA, yellow arrow: hyoid bone).

Approximately 2 months before his first visit to our department, although there were no new symptoms, a repeat head MRI showed hyperintensity on DWI and T2-weighted images, and hypointensity on T1-weighted images in the left frontal, temporal and parietal lobes with a normal MRA. A repeat MRI at our institution showed multiple lesions in the left MCA territory, indicating a convalescent ischemic stroke (Figure 1). A carotid ultrasound in neutral head position showed increased intima-media thickness (IMT) with single plaque formation in bilateral carotid arteries, whereas in the rotated neck position revealed a heterogeneous echo outside the left carotid bifurcation. When the patient was in the right lateral decubitus position with neck rotating to the left, the carotid duplex scan demonstrated a compressed and narrow lumen at the distal end of the left common carotid artery (CCA), which was related to the hyoid bone, and the transcranial color-coded Doppler (TCCD) showed a significantly decreased flow velocity of the left MCA (Figure 2). This phenomenon was not found in the rotation to right. And the compression of hyoid bone to carotid arteries in the right side was not recognized. He subsequently underwent a computed tomography angiography (CTA) of the neck and head, which revealed an enlarged left hyoid bone on close contact with carotid bifurcation, without evidence of artery stenosis (Figure 3). Time of flight-magnetic resonance angiograph (TOF-MRA) showed abnormal signal in the left-internal carotid artery (ICA) lumen, which was more be likely caused by artifact, because the signals of right CCA and bilateral vertebral artery (VA) were also decreased at the same level, as well as the flow on the left ICA on carotid ultrasound on neutral head position was normal. High-resolution magnetic resonance imaging (HR-MRI) displayed no abnormality in the arterial wall. Subsequent DSA revealed no dissection, stenosis or pseudoaneurysm of the internal and external cranial arteries, and no arterial repair or reconstruction was necessary. Extensive workup for multiple causes of stroke in young adults was unrevealing, including complete blood count, erythrocyte sedimentation rate, liver and renal function tests, lipid profile, electrolytes, hemoglobin A1c and C-reactive protein. Thyroid function analysis, tumor markers test, antithrombin III activity, protein C and protein S levels, homocysteine and D-Dimer levels were within the normal limits. Serologic tests of human immunodeficiency virus, hepatitis B and C viruses, syphilis, anti-cardiolipin antibodies, beta-2-glycoprotein antibody, antinuclear antibody and rheumatoid factor were negative. A transthoracic echocardiogram did not demonstrate thrombus, right-to-left shunting post PFO closure or valvular disease, and 24-h Holter monitoring demonstrated episodic bradycardia without atrial fibrillation.

Based on the imaging study as well as the lack of other findings, the working diagnosis was considered as the external compression of the left carotid artery by the hyoid bone, resulting in recurrent ischemic strokes. Given repeated recurrence under antiplatelet treatment, anticoagulation was added. The following 2-year follow-up showed no new neurologic events or any other complaints.

## Discussion

Carotid artery compression by anomalous cervical structures is rare and the mechanical impingement of the carotid vessels related to the bone structures, which eventually results in neurovascular events is even more uncommon. The stylohyoid complex, comprising the styloid process, the stylohyoid ligament and hyoid bone, is in proximity to the ICA (3). In 1937, Eagle first described a phenomenon called “Eagle syndrome” or “stylocarotid artery syndrome,” representing neurologic symptoms caused by compression or irritation of the extracranial carotid artery due to an elongation of the styloid process more than 30 mm and possible ossification of stylohyoid ligament (4). However, rare studies of hyoid bone-related carotid artery disease have been reported.

The hyoid bone, an attachment locus for neck, tongue and throat muscles, is a midline structure in front of cervical spine, below the mandible, above the thyroid cartilage and at the level of the third cervical vertebra. Hyoid bone, consisting of the greater and lesser horns and the body, is horseshoe shaped, which ends anteriorly and superiorly to the carotid artery bifurcation. Fakhry et al. (5) analyzed 180 intact hyoid bones, found that characteristics of the hyoid bone were highly heterogeneous, which were closely associated with the sex, height, and weight of the individuals. In this study, the width of the hyoid bone, meaning the distance between the distal parts of the greater horns of the hyoid bone, was 40.78 ± 7.09 mm. The length of the hyoid bone, presenting the distance from the middle of the anterior part of the body to a hypothetical line connecting the distal parts of the greater horns, was 36.38 ± 4.88 mm. And the width of the body was 20.91 ± 3.04 mm. Duan et al. (6) analyzed 74 intact hyoid bones and found that the length of the right greater horn was 31.4 ± 2.6 mm, of the left was 31.0 ± 2.5 mm.

The anatomy of the hyoid bone is variable and its position changes with swallowing, talking, and neck rotation (7, 8). Commonly, the carotid bifurcations are at the level of C3/4, and the ICA walks posterolaterally to the external carotid artery (ECA). The ICA lesions can be provoked by the movement and impingement of the greater horn of the hyoid bone.

Possible pathological presentations of hyoid bone-related carotid injury include dissection, pseudoaneurysm, stenosis or occlusion due to direct compression, and pressure-induced plaque formation and/or rupture. A possible explanation for carotid vasculopathy is the direct compression of the hyoid bone, leading to constructional changes in the carotid wall and/or an indirect effect via changes in blood flow and shear force. Each of these causes may potentially lead to stenosis, occlusion, or artery-to-artery embolism.

Pearlman et al. (9) reported an 83-year-old man with a suspected stenosis of the left ICA with hemodynamical deficiency. The left carotid endarterectomy revealed a paucity of plaque and obstructive lesion, but a long extension of the hyoid bone impinging on the artery was found, which created significant pressure over the ICA, resulting in a narrow lumen or occlusion of the artery in some certain positions. Previous studies (8–12) described several cases presented as embolic strokes due to chronic trauma to the ICA caused by an elongated hyoid bone, which accelerated plaque accumulation and increased the risk of artery-to-artery embolism. Another case report of a young male patient without any vascular risk factors or relevant family history demonstrated that ICA dissection was related to direct mechanical interference of the ICA by the hyoid bone when rotating or stretching neck (13). In a retrospective multicenter case-control study of carotid artery dissection patients, Renard et al. (14) found that shorter distances between the stylohyoid complex and ICA predispose to the occurrence of carotid artery dissection through mechanical injury. In another case, the patient's transient ischemic symptoms were contributed to the intermittent impingement on the ICA by the elongated hyoid wing, in the absence of atherosclerosis (15). Schneider et al. (16) reviewed three cases of pseudoaneurysm of the carotid artery caused by the mechanical injury of the hyoid bone. The phenomenon of hyoid mechanical trauma to the ICA leading to thrombus formation, embolization and recurrent TIAs, was termed as carotid artery entrapment by the hyoid.

In our case, the width and length of the hyoid bone was 65 mm and 32 mm respectively, the width of the body was 24 mm, and the length of the greater horn was 37.9 mm. We speculate that repetitive mechanical interference to the wall of the carotid artery caused a vessel wall injury with subsequent intimal thrombus formation and cerebral embolization that triggered recurrent neurovascular events. Additionally, luminal obstruction of the carotid artery might disturb the cerebrovascular flow by provoking movements. The hyoid bone movement induced by the extreme and routine neck rotation or stretching may be responsible for the vessel injury. The case reported here calls attention to the initial presentation as TIA followed by a stroke. Despite transcatheter PFO closure and antiplatelet treatment, the patients suffered recurrent cerebrovascular events in the same artery territory. In the third episode, ultrasonography demonstrated variations in blood flow along with the alterations in hyoid positioning in relation to the carotid vessels, and the CTA identified a close contact between the left greater horn of the hyoid bone and the carotid artery.

Due to the heterogeneity of the symptomatology of the Eagle clinical picture and rare anomalies of hyoid bone, it's hard to make the diagnosis. Imaging studies are needed to determine whether compression of the carotid artery by the hyoid bone can occur. Ultrasonography allows to visualize compression of the carotid artery by the hyoid bone on head rotation and swallowing, though requiring considerable experience of the examiner. CTA is considered as an excellent option of diagnosis not only for estimating the length and thickness of the hyoid bone but also for determining its anatomical relationship with blood vessels and muscles, which was less operator dependent. Because CTA is static and lacks dynamic information, dynamic 3D-CTA may be useful for understanding the dynamic anatomical relationship of the carotid arteries with surrounding structures during head rotation and swallowing, which may be used to provide dynamic modalities in future assessment. HR-MRI, as an advanced MRI modality, can render arterial wall and characterize vessel wall pathology. Classic angiography with intra-arterial application of contrast agent and application of functional tests may provide evidence indicating underlying pathophysiology of hyoid bone-related carotid injury such as dissection, pseudoaneurysm, stenosis or occlusion.

In terms of treatment, there are no established treatment guidelines due to the scarcity of reported cases. Treatment varies depending on the pathology (17). Anticoagulation and/or antiplatelet treatment is usually initiated (8). When an artery-to-artery embolism is suspected or in cases of ICA dissection, anticoagulation therapy in the early phase can be considered in the absence of contraindications. Surgical partial bone resection, proved to be safe and effective (10), remains a viable option especially in cases of repeated recurrence under medication treatment, which restores the patient with physical and psychological freedom of neck movement. For our patient, there was no evidence of focal carotid stenosis, leading to a speculation of artery-to-artery embolism which was attributed to continued clot formation caused by localized trauma. We initiated anticoagulation therapy, and the patient remained free of ischemic event in the following 24 months.

Our study has several limitations. First, we didn't check whether the hyoid bone compress the carotid artery during swallowing or neck flection and lacks dynamic information. Second, due to the limited cases, there has been no established strategy in cases of hyoid bone compression-related embolic stroke. Although the patient remained free from ischemic events following 2 years, surgical resection should be recommended in this case. And long-term follow-up should be conducted.

## Conclusions

Hyoid bone-related carotid artery injury is a rare etiology of stroke, which is unknown to many doctors, thus itis not considered in the differential diagnosis of the cause of a stroke. For patients with recurrent strokes or TIAs, especially in young patients, consideration of mechanical compression and trauma of the vessels should be entertained, if work-up for common etiologies reveals no clear cause. Carotid artery impingement with compression by the hyoid bone seems to be extremely uncommon and a diagnosis of exclusion. Imaging studies with provocative maneuvers are helpful to make the diagnosis. In addition, the optimal treatment remains unclear. The cases presented here (6, 7, 9, 11–13, 15–27) contribute to further delimitation of the clinical and radiological diagnostic criteria, exploratory findings, and management of hyoid impingement.

## Data Availability Statement

The original contributions presented in the study are included in the article/Supplementary Material, further inquiries can be directed to the corresponding author/s.

## Ethics Statement

The studies involving human participants were reviewed and approved by the Ethics Committee of Xuanwu hospital. The patients/participants provided their written informed consent to participate in this study. Written informed consent was obtained from the individual(s) for the publication of any potentially identifiable images or data included in this article.

## Author Contributions

GL and YW: drafting of the manuscript. GL, YW, and HS: concept and design. YW: acquisition, interpretation of data and obtained funding. HS and XJ: critical revision of the manuscript for important intellectual content. CC, YR, and YH: administrative, technical or material support. All authors contributed to the article and approved the submitted version.

## Conflict of Interest

The authors declare that the research was conducted in the absence of any commercial or financial relationships that could be construed as a potential conflict of interest.

## Publisher's Note

All claims expressed in this article are solely those of the authors and do not necessarily represent those of their affiliated organizations, or those of the publisher, the editors and the reviewers. Any product that may be evaluated in this article, or claim that may be made by its manufacturer, is not guaranteed or endorsed by the publisher.
