# Supplementary material for: Hyoid Elongation May Be a Rare Cause of Recurrent Ischemic Stroke in Youth-A Case Report and Literature Review
Source: Front Neurol. 2021 Sep 1;12:653471. doi: 10.3389/fneur.2021.653471 (PMC8440883; doi:10.3389/fneur.2021.653471)
Supplement: Supplementary file 1 [file Table_1.DOCX]

Supplementary Table 1. Summary of hyoid-related carotid artery diseases from 1999 to 2020

| References | Age at diagnosis (years) / sex | Predisposing factors | Clinical presentation | Brain imaging | Cerebrovascular imaging | Surgery findings | Treatment | Outcome |
| --- | --- | --- | --- | --- | --- | --- | --- | --- |
| [Abdelaziz](https://pubmed.ncbi.nlm.nih.gov/?term=Abdelaziz+OS&cauthor_id=10369234)  et al^11^, 1999 | 85/F | None | Left hemiparesis | CT showed no infarction. | MRA showed 90% stenosis of the right ICA | The hyoid bone projected into the internal carotid artery producing a significant indentation in the vessel. | Partial hyoid bone resection. | With no untoward effects |
| Schneider et al^16^, 2007 | 47/M | Carried heavy load on his left shoulder. | A pulsatile mass on the left neck. | CT showed a spheroid-shaped hematoma in the soft tissues of the left neck. Fresh blood with close contact to the carotid bifurcation was seen in the central part of the hematoma. | The CCA below the bifurcation was found to be the source of bleeding by three-dimensional reconstruction of the CT scan. | Intraoperative finding confirmed a pseudoaneurysm originating from a perforation of the distal CCA. There was a close relationship between the arterial wall and the greater horn of the hyoid bone, which was palpable in the depth of the aneurysm.  Histopathologic examination of the resected artery segment showed chronic inflammatory infiltrates and an increase of connective tissue corresponding to ongoing mechanical trauma, without arteriosclerotic changes. | Resection of the perforated segment of the CCA and the greater horn of the hyoid bone. | Unknown. |
| Kölbel et al^15^, 2008 | 61/F | A sudden pressure in her head and chest. | Transient left hemiparesis. | CT showed no signs of bleeding or ischemic infarction. | UDS revealed an ICA stenosis on the symptomatic right side of about 55%. CTA showed that the right greater cornu of the hyoid bone interposed between the ICA and ECA. | The segment of the ICA in contact with the hyoid bone appeared intraoperatively ectatic but otherwise normal and soft, without an apparent perivascular fibrosis. | Resection of the right greater cornu of the hyoid bone. | No neurologic symptoms for 1 year. |
| Hong et al^12^, 2011 | 56/M | Playing golf. | Numbness or weakness of left limbs. | MRI showed acute scattered infarcts in the right MCA territory and old infarcts on the border zone in the right hemisphere. | CTA showed ICA adjacent to the greater horn of the right hyoid bone. | None | Partial hyoid bone resection. | Remained symptom free for 6 months. |
| Mori et al^17^, 2011 | 61/M | None | Aphasia, right hemiparesis and retinal ischemia. | CT showed an ischemic lesion in the left basal ganglia. | UDS showed a thrombus-like entity in left ICA.  CTA showed the greater horn of hyoid bone compressing the narrowest segment of the left ICA from behind. | DSA showed occlusion of the ICA 1 year before admission, recanalized, with severe segmental stenosis on day 13.  A pathological examination of the arterial wall tissue showed only fibrotic change. | Adhesiotomy from the circumferential tissues and patch formation of the left ICA. combined with antiplatelet therapy. | Recovered without sequelae. |
| Renard  et al^18^, 2011 | 83/M | Repetitive head rotation in normal daily life. | Acute transient sensory deficit without pain of right hand while eating. | MRI showed A chronic (asymptomatic) watershed infarction between the left PCA and MCA without recent infarction. | DUS showed a large atheromatous plaque at the proximal ICA with an irregular surface, associated with a 50% stenosis. Velocities did not change after head rotation.  CTA confirmed focal vasculopathy in the antero-medial side of the ICA, in near contact with the greater horn of the left hyoid bone. | None. | Anticoagulation, switched to acetylsalicylic acid after 3 months | Unknown |
| Renard  et al^19^, 2012 | 30/M | Left heavy weight. | Anterior neck pain at the right side and left hemiplegia. | MRI showed an acute infarction in the right MCA territory. | DUS revealed a high degree of stenosis of the right ICA.  CTA showed a segmental tapered narrowing of the right ICA located just lateral of the greater horn of hyoid bone, with an elongated styloid process, without atheromatous plaques.  Fat-saturated T1 MRI showed an intramural hematoma in the right ICA. | None | Unknown | CTA revealed complete recanalization of the right ICA. DUS showed normal velocities, without changes in velocities after head rotation. |
| Pearlman et al^9^, 2012 | 83/F | None. | Right hemiparesis. | None | DUS revealed carotid stenosis greater than 70% on the left side with hemodynamical deficiency.  CTA showed protrusion of the hyoid bone into the carotid artery. | No significant obstructive lesion was found, but the arterial wall of ICA opposite the hyoid bone was bruised and the intima had formed a web projecting from the wall of the vessel. | Carotid endarterectomy. | No recurrent episodes of TIAs or symptoms of carotid insufficiency for several months. |
| Janczak  et al^7^, 2012 | 36/M | Turning head to the left. | Recurrent syncope or near-syncope episodes often preceded by visual symptoms. | None | DUS revealed critical stenosis or even complete closure of the ICA with head rotation to the right.  CTA revealed an anatomical anomaly of the hyoid bone with elongated lesser cornu，which was very close to the right ICA. | None | Resection of the elongated smaller horn. | No recurrence during 2-year follow-up. |
| Yukawa et al^13^, 2014 | 36/M | Neck rotation or stretching. | Left hemiparesis and anterior neck pain at the right side. | MRI showed acute infarction in the right frontal lobe. | MRA showed occlusion of the right ICA.  CTA showed the right greater horn of the hyoid bone adjacent to the right ICA | DSA showed tapered occlusion of the right ICA, suggesting carotid artery dissection. | Aspirin and edaravone. | Discharged with minimal neurologic deficit 20 days later |
| Tokunaga et al^20^, 2015 | 46/F | None | Repeated episodes of transient left hemiparesis and sensory disturbance. | MRI showed infarcts in the right MCA territory. | DUS showed the origin of the right ICA moving dynamically according to head rotation and swallowing and there was a mural thrombus at the origin of the right ICA.  Cervical T1-weighted MRI revealed that the origin of the right ICA was hooked by the right greater cornu of the hyoid bone when it became stuck. | Intraoperatively, the vessel wall of the origin of the right ICA was thickened with ulceration. Microscopically, the specimen obtained by carotid endarterectomy demonstrated medial necrosis with inflammatory changes and mural organized thrombus without atherosclerosis. | Partial resection of the right greater cornu of the hyoid bone and carotid endarterectomy. | Remained free of ischemic attack over 6 months after operation. |
| Kinoshita et al^21^, 2017 | 70/M | Swallowing | Amaurosis fugax of the right eye | MRI showed an acute asymptomatic brain infarct in the right frontal lobe cortex. | DUS showed movement of the right ICA, with a hypoechoic plaque at its origin, in contact with the hyoid bone during head rotation and swallowing. Dynamic MRI allowed visualization of ICA migration during swallowing. | None | Unknown | Unknown |
| Ludt, et al^6^, 2018 | 32/F | None | Episodic left-sided numbness and weakness. | MRI showed small areas of acute infarction of the right frontal and parietal lobes. | CTA showed a small thrombus in the right carotid bifurcation with extension into the proximal ICA. The greater cornu of the hyoid bone elongated bilaterally, with the right horn extending between the right ECA and ICA | None | Hyoid bone resection, combined with aspirin. | Stroke symptoms have not recurred. |
| Campos  et al^22^, 2018 | 29/M | Long-time hiking with a backpack. | Paralysis of the left upper limb. | MRI showed acute infarction in the cortico-subcortical boundary of the right parietal lobe. | CTA depicted an enlarged greater horn of the hyoid bone, closely contacted with the ICA, where a hypoattenuating irregularity on the parietal wall was found, accompanied by a tiny flap of the endothelium consistent with a thrombus. | Evident atherosclerosis and a small intimal thrombus adjacent to the compression site were seen at the opening of the arterial segment.  The microscopic examination showed focal intimal thickening with fibrosis, and an accumulation of foam cells-typical of uncomplicated atherosclerotic plaques, without inflammatory infiltration. | Resection of the posterior horn of the hyoid bone, and the injured segment of the ICA, followed by carotid–carotid bypass with the great saphenous vein. | NO new events or any other complaint during the 5-month follow-up. |
| [Yamaguchi](https://pubmed.ncbi.nlm.nih.gov/?term=Yamaguchi+Y&cauthor_id=32642020) et al^23^, 2018 | 63/F | Swallowing | Consciousness disturbance and right hemiparesis, combined with fever and left neck pain. | MRI revealed multiple hyperintensities in the left frontal and parietal lobes. | DUS showed calcified plaque with vessel wall swelling at the bifurcation of the left CCA and surrounding hypoechoic soft tissue. DUS also revealed that the left CCA transposed to the retropharyngeal position with swallowing and was compressed by the hyoid bone. | None | Corticosteroid therapy | Symptoms and radiological findings improved |
| Martinelli et al^24^，2019 | 61/M | Prolonged rotation of his head. | TIA with left-sided hemiparesis. | CT showed no intracranial hemorrhage. | DUS showed an iso-echoic, fibrotic plaque of the right ICA which presented an anomalous course at this level, suggesting severe right carotid stenosis  CTA showed right greater horn of the hyoid bone was interposed between the ipsilateral ICA and ECA | Macroscopic observation found a fibrous atheroma and ICA wall thickening. The pathological examination confirmed the atheromatous nature of the arterial lesion. | Endarterectomy | No recurrence. |
| Plotkin et al^10^, 2019 | 32/F | None | Left-sided numbness after cerebrovascular accidents, multiple episodes of dysphasia and left-sided weakness almost daily | MRI showed small acute infarcts in the right MCA distribution, indicating a thromboembolic etiology. | DUS confirmed that the hyoid bone was located between the ICA and ECA in a neutral position. With neck rotation, the hyoid repositioned laterally, slipping across the ICA and out of the bifurcation.  CTA revealed the an enlarged right hyoid bone located between the ICA and ECA. There was also a residual nonocclusive thrombus along the medial aspect of the right carotid. bifurcation with extension into the right ICA | None | A short course of anticoagulation and antiplatelet therapy to resolve the thrombus and partial surgical resection of the hyoid. | Full resolution of neurologic function with no further neurologic events at 12 months postoperatively. |
| Kho et al^25^, 2019 | 29/F | Rapid rotation from left to right. | Intermittent episodes of expressive dysphasia, right arm weakness. | MRI showed multiple infarcts in left MCA and ACA territories | CTA demonstrated that minor irregularities of the proximal left ICA, and the ICA was postero-medial to ECA and behind the greater cornu of the hyoid | DSA showed medial subluxation of the left ICA to behind the greater cornu of the hyoid bone on extreme left lateral head tilt. | Aspirin. | Stroke recurrent in the same territory five months later. |
|  |  |  |  |  |  |  | Resection of the left greater cornu of the hyoid. | No recurrent events. |
|  | 34/F | A constant right earache that previously had been intermittent for two years. | Dysarthria and left facial droop with a sudden onset. | MRI showed multiple infarcts in the right frontal lobe | CTA showed a shelf-like protuberance from the medial wall of the right carotid artery, just above the carotid bifurcation. The right ICA was subluxed medially in the neutral position, but upon right head turning, it flipped to its usual expected position. The right ICA showed eccentric medially-directed intimal thickening. | None | Aspirin | Ear pain persisted but no new strokes In the following 12 months. |
|  |  |  |  |  |  |  | Resection of right greater cornu of the hyoid bone. | Resolution of right ear pain and no further neurological issues. |
| Liu et al^26^, 2020 | 81/M | None | An episode of left facial and upper extremity paresthesia and paresis. | MRI showed cortical infarcts within the right precentral and postcentral gyri | DUS demonstrated moderate to severe atherosclerotic disease at the right carotid bulb.  CTA showed proximity of the hyoid bone to the carotid bulb | DSA showed an eccentric, focal stenosis of the carotid bulb. Histologic evaluation of the plaque demonstrated calcified atheroma. | Carotid endarterectomy. | Remains without hemodynamically significant stenosis at 15 months of follow-up |
| [Yamaguchi](https://pubmed.ncbi.nlm.nih.gov/?term=Yamaguchi+Y&cauthor_id=32642020) et al^27^, 2020 | 76/M | Swallowing | Visual loss in right eye | MRI showed asymptomatic acute infarctions in the right MCA territory. | On DUS, the proximal right ICA was not visualized due to the acoustic shadow from the hyoid bone. 3D-CTA revealed a stenosis of 50% with calcified plaques in the proximal right ICA, which was close to the right greater horn of the hyoid bone and superior horn of the thyroid cartilage, and was compressed by the hyoid bone and thyroid cartilage during swallowing. |  | Partial resection of the hyoid bone and thyroid cartilage, combined with antithrombotic therapy. | No recurrent events. |

Abbreviations: CCA: common carotid artery; CT: computed tomography; CTA: computed tomography angiography; ECA: external carotid artery; ICA: internal carotid artery; MCA: middle cerebral artery; MRA: magnetic resonance angiography; MRI: magnetic resonance imaging; TIA: transient ischemic attacks; DUS: doppler ultrasound spectrum
